# Erythropoietin-producing tubercle granuloma in a hemodialysis patient

**DOI:** 10.1186/1471-2369-14-91

**Published:** 2013-04-21

**Authors:** Minoru Satoh, Hiroshi Ueta, Takehiko Tokura, Tamaki Sasaki, Naoki Kashihara

**Affiliations:** 1 Department of Nephrology and Hypertension, Kawasaki Medical School, 577 Matsushima, Kurashiki, Okayama, 7010192, Japan; 2 Current address; Department of Anesthesiology, Kobe City Medical Center General Hospital, 2-1-1 Minatojima-nakamachi, Chuo-ku, Kobe, Hyogo, 6500046, Japan; 3 Current address; Department of Internal Medicine, Fukuyama City Hospital, 5-23-1, Zao-cho, Fukuyama, Hiroshima, 7218511, Japan

**Keywords:** Erythropoietin, Tubercle granuloma, Hemodialysis, Fever of unknown etiology, In situ hybridization

## Abstract

**Background:**

We describe a case of a fever of unknown etiology that was caused by a caseating tubercle granuloma which produced erythropoietin. To our knowledge, this is the first report of an erythropoietin- producing granuloma.

**Case presentation:**

A 48-year-old Japanese man with a 5-year history of maintenance hemodialysis for diabetic nephropathy presented with an intermittent fever over a few months. During febrile periods he developed erythema nodosum on his legs. Computed tomography showed axillary lymph node enlargement and this was further corroborated by a gallium scan that revealed high gallium uptake in these nodes. A Mantoux test was positive and an interferongamma release assay for tuberculosis diagnosis was also positive. Lymph node tuberculosis was suspected and the patient underwent lymphadenectomy. Histological analysis of the lymph nodes revealed a caseating granuloma that showed positive results on an acid-fast bacteria stain and a Mycobacterium tuberculosis polymerase chain reaction test. After lymphadenectomy, however, the patient’s hemoglobin levels rapidly decreased from 144 to 105 g/L, and this was further compounded by a decrease in serum erythropoietin from 223 mIU/mL to 10.7 mIU/mL by postoperative day 21. We suspected the tubercle to be a source of the erythropoietin and this was further confirmed by in situ hybridization.

**Conclusions:**

We report for the first time ectopic erythropoietin production by a tuberculous lymph node. Our observations are substantiated by a postoperative decline in his erythropoietin level and a clinical requirement for erythropoietin treatment.

## Background

The complication of polycythemia has been described with several malignancies, such as renal cell carcinoma, cerebral meningioma, and hepatocellular carcinoma [1]. In some cases, ectopic erythropoietin (Epo) production has been demonstrated in the tumor [2-8]. However, few reports describe the ectopic production of Epo in benign diseases. Patients with a benign cystic lesion have been shown to have increased Epo production [9,10], but Epo has not been reported as produced ectopically in a lymph node. Here, we describe a dialysis patient who presented with a fever of unknown etiology that was caused by a caseating tubercle granuloma. The caseous tubercle granuloma had produced Epo that resulted in a maintained hemoglobin level in spite of dialysis patient with evidence of inflammation. To the best of our knowledge, this is the first description of an Epo-producing tubercle granuloma.

## Case presentation

A 48-year-old Japanese man with a 5-year history of maintenance hemodialysis for diabetic nephropathy presented with spikes of fever every few months accompanied by the appearance and regression of multiple painful subcutaneous nodules over his legs and feet. These were diagnosed as erythema nodosum by a biopsy examination. Investigations showed had high levels of C-reactive protein (150 to 200 mg/L), a hemoglobin level of 146 g/L, a white blood cell count of 7.0 × 10^9^/L, with 92% neutrophils and a platelet count of 2.2 × 10^11^/L, indicating a lack of anemia in spite of chronic inflammation. Administration of broad-spectrum antibiotics failed to generate a response. Peripheral lymph nodes were not palpable. The patient was also found to be negative for human immunodeficiency virus (HIV) infection. Sputum examination revealed no significant pathogenic bacteria, and an acid-fast stain test and a *Mycobacterium tuberculosis* polymerase chain reaction (PCR) test were both negative. Blood and urine cultures were also negative, and renal ultrasonography did not show renal cysts or masses. Several tests for collagen disorders including vasculitis were all negative.

**Figure 1:**
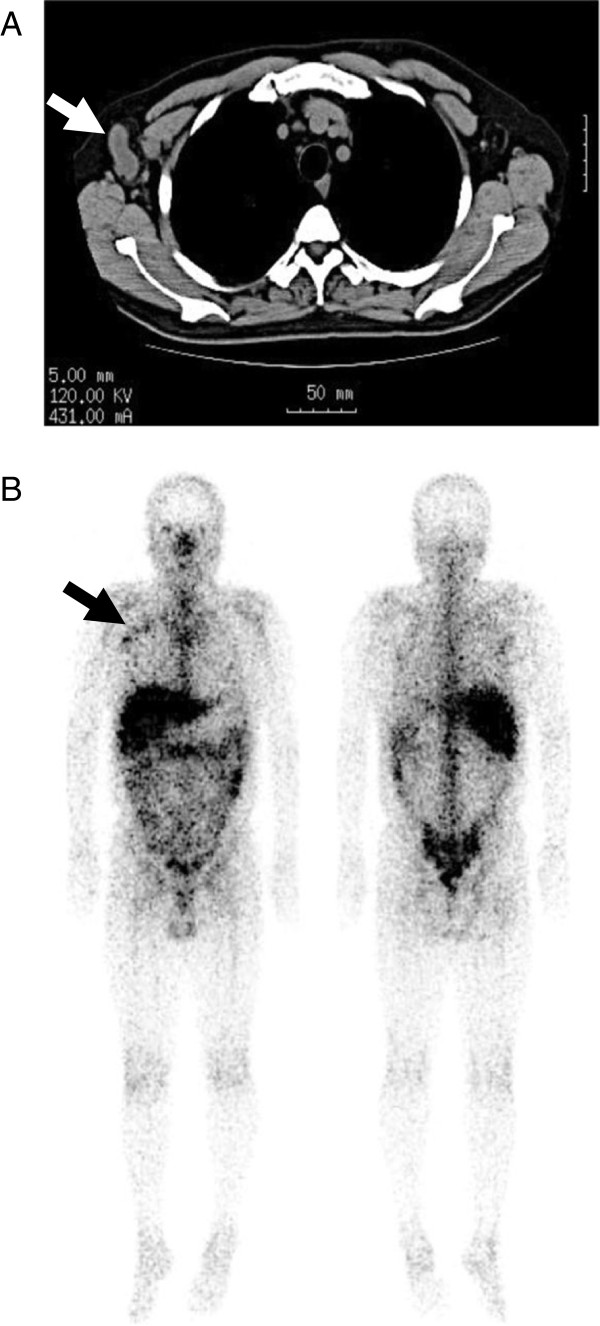
**Images of right axillary lymph node tuberculosis.** Images of computed tomography (**A**) and gallium scanning (**B**). (**A**) Non-enhanced computed tomography scan showing enlarged right axillary lymph nodes (arrow). (**B**) Gallium scan showing abnormal uptake in axillary lymph nodes (arrow).

**Figure 2:**
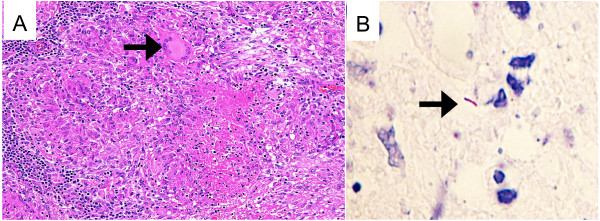
**Pathological images of tubercle granuloma.** Pathological findings (**A**) and acid-fast bacteria stain (**B**) of resected axillary lymph node. (**A**) Histopathological findings of the resected specimen include caseous necrosis and an epithelioid granuloma with Langhans giant cells (arrow) (PAS staining, ×100). (**B**) Microphotograph of the resected axillary lymph node stained with Ziehl-Neelsen stain (×400) showing acid-fast stain-positive bacilli within the tissue (arrow).

Computed tomography showed axillary lymph node enlargement (Figure 1A), which was corroborated by a gallium scan that revealed abnormally high gallium uptake in the axillary lymph nodes (Figure 1B). When administered the Mantoux test, the patient was found to be purified protein derivative, slightly positive at 10 mm, which points to tuberculosis infection. Tuberculosis was further indicated by a positive interferon-gamma release assay. Chest radiography revealed no abnormal shadowing in the lung fields, thus ruling out pulmonary tuberculosis. Based on these results, we suspected lymph node tuberculosis, and axillary lymphadenectomy was performed. Six lymph nodes were surgically removed and their histological examination revealed caseating granulomata in all of the lymph nodes (Figure 2A). Langhans giant cells were also observed in the granuloma. Further, the granuloma tested positive on an acid-fast bacteria stain (Figure 2B) and a *M*. *tuberculosis* PCR test. He was treated with rifampicin, isoniazid, and ethambutol.

**Figure 3:**
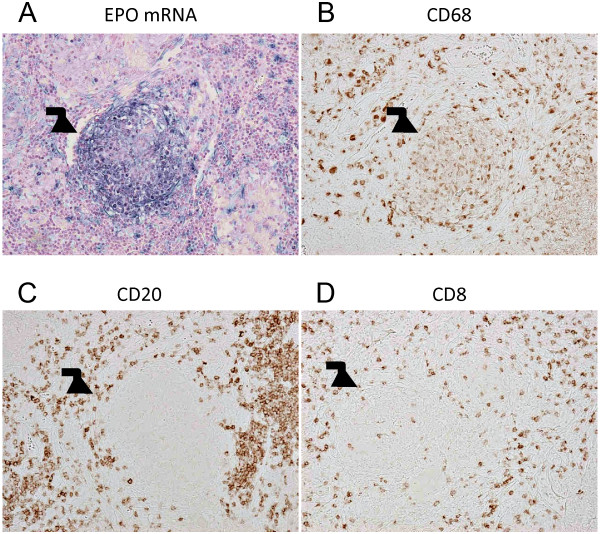
**Epo expression in tubercle granuloma.***In situ* RNA hybridization of the resected axillary lymph node (**A**) and immunohistochemistry for CD68 (**B**), CD20 (**C**), and CD8 (**D**). (**A**) In situ hybridization revealing the expression of Epo mRNA in non-epithelioid cells in lymph nodes. Methods are described in the Additional file 1. Positive signals were observed in the granuloma (arrow) (×200). (**B**) CD68-positive cells were observed in the granuloma (arrow) (×200). (**C**, **D**) CD20- and CD8-positive cells were rarely observed in the granuloma (arrow) (×200).

To our surprise, lymphadenectomy was followed by a rapid decline in the patient’s hemoglobin levels from 144 to 105 g/L. Serum Epo levels also decreased from 223 mIU/mL to 10.7 mIU/mL (upper normal limit 23.7 mIU/mL for normal adult) by postoperative day 21. The patient received a weekly injection of 40 mg iron sucrose, and his serum ferritin level was maintained at 50–150 ng/mL. The patient at this stage needed Epo infusions to maintain hemoglobin levels (target hemoglobin between 100 and 120 g/L). The strong association between the excision of the tubercle and the drop in Epo levels made us examine the granuloma for Epo expression. The tubercle granuloma was found to be positive for Epo on in situ hybridization (Figure 3A). Methods of in situ hybridization are described in the Additional file 1. We further validated this by immunohistochemical staining, wherein strong cytoplasmic staining for Epo was found in these tubercle granuloma cells. The Epo-producing cells were positive for CD68 (monocyte/macrophage marker) but not for CD20 (B lymphocyte marker) or CD8 (cytotoxic T lymphocyte marker) (Figure 3B-D).

## Conclusions

We present here the first known instance of a tubercle granuloma implicated in ectopic Epo production. Ectopic production of a hormone or cytokine by a tumor is well recognized but not common. Ectopic Epo production leading to polycythemia has been previously described in association with several malignancies [1]. Reports have demonstrated local existence and production of Epo in the tumor mass in several different cancer types such as hepatocellular carcinoma [2,3], renal cell carcinoma [4,5], gastric carcinoma [6], thymic carcinoma [7], and lung adenocarcinoma [8].

In our case study, the patient was on chronic maintenance dialysis, and this complicated the diagnosis. Although patients with end stage renal disease undergoing chronic dialysis are much more susceptible to tuberculosis than the general population, the diagnosis is often difficult because of frequent extrapulmonary involvement and nonspecific symptoms [11]. Extrapulmonary tuberculosis accounts for approximately 15% to 20% of tuberculosis cases in immunocompetent patients [12]. Because tuberculosis is commonly responsible for fevers of unknown origin [13], we recommend that a systematic diagnosis approach [14] be used in the diagnosis of a fever of unknown etiology in patients receiving dialysis. We believe that a thorough systematic differential diagnosis approach will allow comprehensive examination of the patient including an examination of the lymph nodes and may thus minimize the possibility of overlooking tuberculosis. Chronic infections like tuberculosis are usually accompanied by anemia because of lowered Epo production caused by cytokines such as tumor necrosis factor [15]. The occurrence of polycythemia instead of anemia in a patient with chronic tuberculosis is a rare event. In our patient, we believe that the expected anaemia was avoided because of the Epo overproduction by the granuloma.

While anemia is common in patients on chronic hemodialysis, spontaneous erythrocytosis is rare. In rare cases, hydronephrotic kidney disease [16], obstructive sleep apnea [17], or renal artery thrombosis [18] can cause Epo-dependent secondary polycythemia in a dialysis patient. A high serum Epo level in polycythemic patients suggests secondary erythrocytosis as a possible diagnosis. In the present case, the hemoglobin data of the patient were not indicative of erythrocytosis. However, we measured Epo levels because hemoglobin levels were relatively high despite the presence of chronic inflammation, which normally suppresses erythropoiesis. We believe that serum Epo concentration should be checked to detect any possible instances of secondary erythrocytosis whenever the hemoglobin level is disproportionate with the clinical condition. Particularly in cases like ours, where the patient was on maintenance dialysis, we speculate that the renal anemia due to chronic dialysis was able to mask the effects of polycythemia caused by the Epo over-production.

Epo belongs to a family of non-immunological cytokines [19] and is produced by fibroblasts derived from the neural crest in renal interstitial spaces [20]. In the present case, Epo mRNA was observed primarily in the CD68-positive area of the caseous tubercle granuloma. A previous report indicated that macrophages can potentially produce Epo extrarenally [21]. Epo expression is known to be upregulated by hypoxia through a transcription factor hypoxia-inducible factor-1 [22]. Other transcription factors like Wilms tumor protein, Wt1 [23] and GATA-4 [24] are also involved in Epo gene expression in the liver. So, in this light, although the molecular mechanism underlying Epo production in macrophages is not known, it is not an implausible finding.

In conclusion, we report, to the best of our knowledge, the first case of Epo-producing tubercle granuloma in a hemodialysis patient. The postoperative clinical exacerbation of anemia and the decrease in Epo levels corroborate our finding and suggest a diagnosis of an Epo-producing granuloma. We speculate that a previously unknown mechanism of Epo production must be operative in this tubercle granuloma.

## Consent

Written informed consent was obtained from the patient for publication of this case report and any accompanying images. A copy of the written consent is available for review by the Editor of this journal.

## Abbreviations

Epo: Erythropoietin

## Competing interests

The authors declare that they have no competing interests.

## Authors’ contributions

MS participated in the histological review of tissues and prepared the final version of the manuscript. KU and TT were involved in the clinical follow-up of the patient. TS contributed to the discussion of the conclusions. NK contributed to the final preparation of the manuscript. All authors have read and approve the final manuscript.

## Pre-publication history

The pre-publication history for this paper can be accessed here:

http://www.biomedcentral.com/1471-2369/14/91/prepub

## Supplementary Material

Additional file 1: Figure S1 *In situ* RNA hybridization of the resected axillary lymph node using erythropoietin antisense and sense probes. The tubercle granuloma was found to be positive for Epo on *in situ* hybridization. Click here for file
