# Supplementary material for: Erythropoietin-producing tubercle granuloma in a hemodialysis patient
Source: BMC Nephrol. 2013 Apr 21;14:91. doi: 10.1186/1471-2369-14-91 (PMC3646680; doi:10.1186/1471-2369-14-91)
Supplement: Additional file 1: Figure S1 — In situ RNA hybridization of the resected axillary lymph node using erythropoietin antisense and sense probes. The tubercle granuloma was found to be positive for Epo on in situ hybridization. [file 1471-2369-14-91-S1.doc]

**Methods**

**In situ hybridization**

Paraffin embedded blocks of human lymph tissues were sectioned at 6µm for *in situ* hybridization. The sections were de-waxed with xylene, and rehydrate through an ethanol series and PBS. The sections were fixed 4% para-formaldehyde in PBS for 15min and then washed with PBS. The sections were treated with 8 µg/ml ProteinaseK in PBS for 30min at 37˚C, washed with PBS, re-fixed with 4% para-formaldehyde in PBS, again washed with PBS, and placed in 0.2N HCl for 10min. After washing with PBS, the sections were acetylated by incubation in 0.1 M tri-ethanolamine-HCl, pH 8.0, 0.25% acetic anhydride for 10min. After washing with PBS, the sections were dehydrated through a series of ethanol. Hybridization was performed with probes at concentrations of 300 ng/ml in the Probe Diluent-1 (Genostaff, Tokyo, Japan) at 60˚C for 16hr. After hybridization, the sections were washed in 5x Hybri Wash (Genostaff), equal to 5x SSC, at 60˚C for 20min and then in 50% formamide, 2x Hybri Wash at 60˚C for 20min, followed by RNase treatment in 50 µg/ml RNaseA in 10 mM Tris-HCl, pH 8.0, 1 M NaCl and 1 mM EDTA for 30min at 37C. Then the sections were washed twice with 2x Hybri Wash at 60˚C for 20min, twice 0.2x Hybri Wash at 60˚C for 20min, and once with TBST (0.1% Tween20 in TBS). After treatment with 0.5% blocking reagent (Roche Diagnostics Japan, Tokyo, Japan) in TBST for 30min, the sections were incubated with anti-DIG AP conjugate (Roche) diluted 1:1000 with TBST for 2hr at RT. The sections were washed twice with TBST and then incubated in 100 mM NaCl, 50 mM MgCl_2_, 0.1% Tween20, 100 mM Tris-HCl, pH9.5. Coloring reactions were performed with NBT/BCIP solution (Sigma-Aldrich Japan, Tokyo, Japan) overnight and then washed with PBS. The sections were counterstained with Kernechtrot stain solution (Mutoh, Tokyo, Japan), dehydrated, and then mounted with Malinol (Mutoh) and observed by the BX43 microscopy (Olympus, Tokyo, Japan). The typical staining was shown in Additional Figure 1.

**Probe information**

Probe: Epo

human Erythropoietin (Epo), Accession# NM_000799, Sequence Size: 1340

Sequence Position: 216-619, Size: 404

TTCTCCTGTCCCTGCTGTCGCTCCCTCTGGGCCTCCCAGTCCTGGGCGCCCCACCACGCCTCATCTGTGACAGCCGAGTCCTGGAGAGGTACCTCTTGGAGGCCAAGGAGGCCGAGAATATCACGACGGGCTGTGCTGAACACTGCAGCTTGAATGAGAATATCACTGTCCCAGACACCAAAGTTAATTTCTATGCCTGGAAGAGGATGGAGGTCGGGCAGCAGGCCGTAGAAGTCTGGCAGGGCCTGGCCCTGCTGTCGGAAGCTGTCCTGCGGGGCCAGGCCCTGTTGGTCAACTCTTCCCAGCCGTGGGAGCCCCTGCAGCTGCATGTGGATAAAGCCGTCAGTGGCCTTCGCAGCCTCACCACTCTGCTTCGGGCTCTGGGAGCCCAGAAGGAAGCCATC

Epo antisense

Epo sense


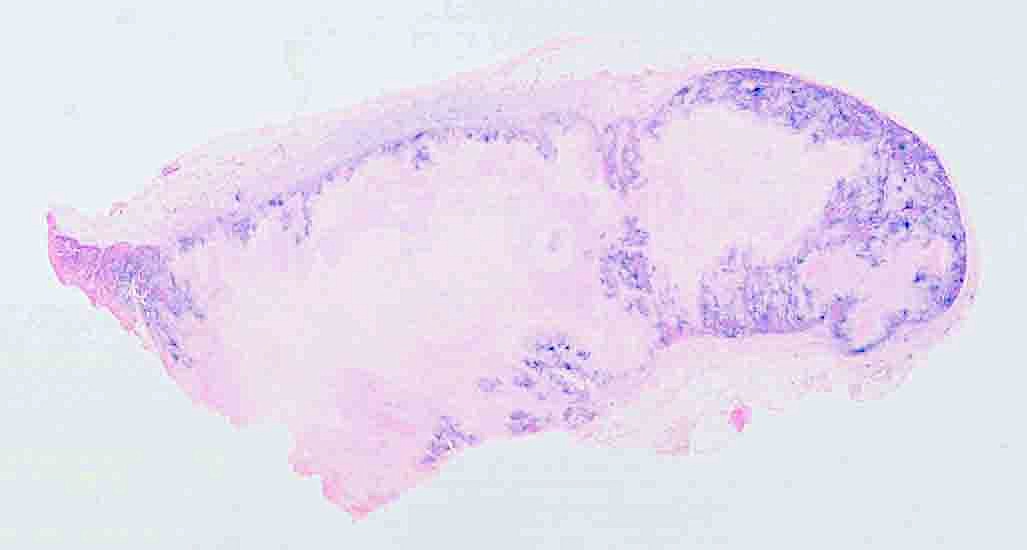

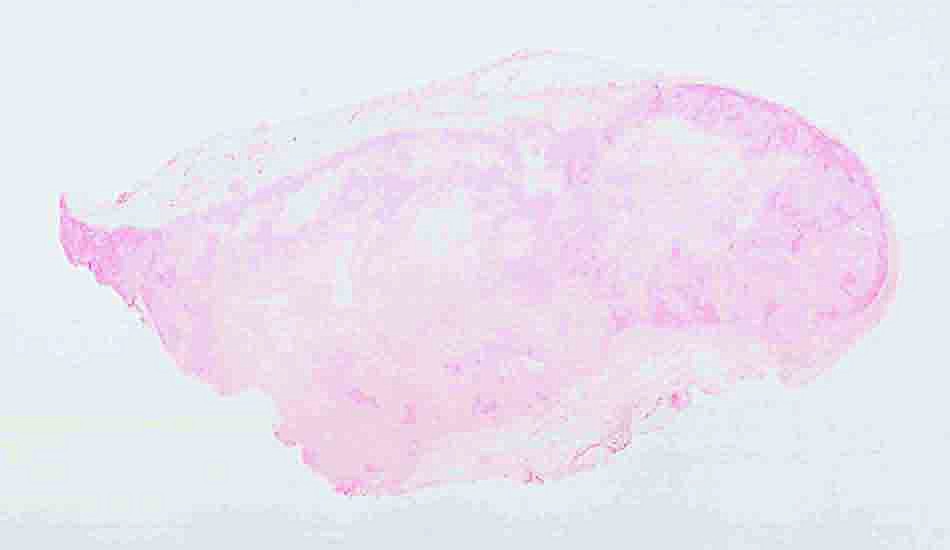

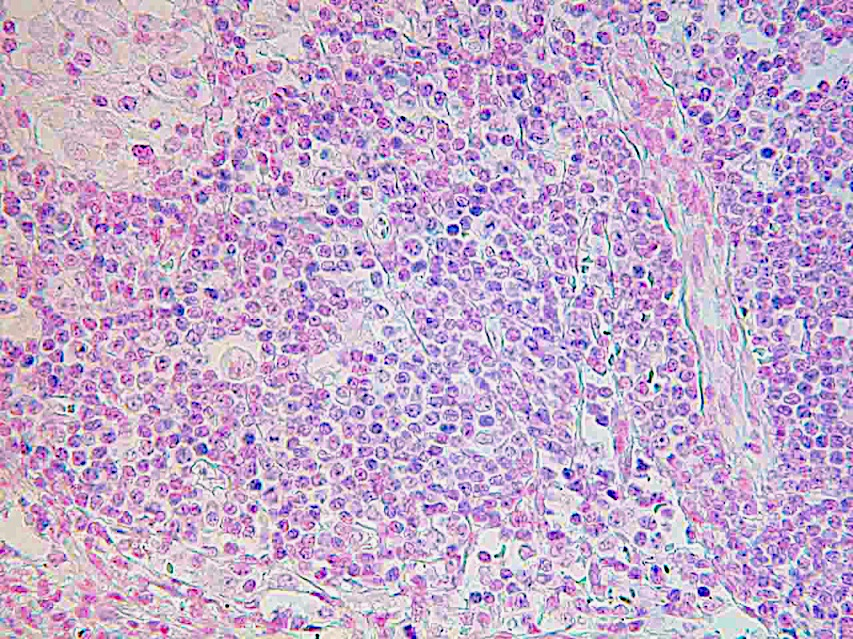

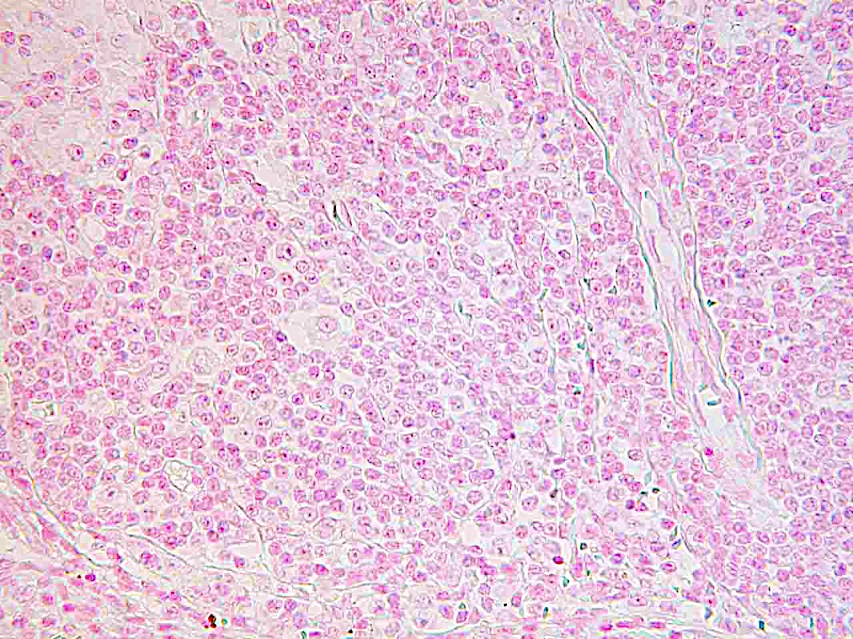


**Additional Figure 1 *In situ* RNA hybridization of the resected axillary lymph node using erythropoietin antisense and sense probes.** The tubercle granuloma was found to be positive for Epo on *in situ* hybridization.
